# Molecular Cloning and Characterization of the First Caspase in the Striped Stem Borer *Chilo suppressalis*

**DOI:** 10.3390/ijms140510229

**Published:** 2013-05-15

**Authors:** Ming-Xing Lu, Yu-Zhou Du, Shuang-Shuang Cao, Pingyang Liu, Jianyong Li

**Affiliations:** 1 College of Horticulture and Plant Protection & Institute of Applied Entomology, Yangzhou University, Yangzhou 225009, Jiangsu, China; E-Mails: lumingxing0123@yahoo.com.cn (M.-X.L.); caosshuang@163.com (S.-S.C.); 2 Department of Biochemistry, Virginia Tech, Blacksburg, VA 24061, USA; E-Mail: lpingyang1987@gmail.com

**Keywords:** apoptosis, *Cs*caspase-1, *Chilo suppressalis*, expression, development, thermotolerance

## Abstract

Apoptosis is executed through the activity of the caspases that are aspartyl-specific proteases. In this study, we isolated the caspase gene (*Cs*caspase-1) of *Chilo suppressalis* (one of the leading pests responsible for destruction of rice crops). It possesses the open reading frame (ORF) of 295 amino acids including prodomain, large subunit and small subunits, and two cleavage sites (Asp^23^ and Asp^194^) were found to be located among them. In addition to these profiles, *Cs*caspase-1 contains two active sites (His^134^ and Cys^176^). Genomic analysis demonstrated there was no intron in the genome of *Cs*caspase-1. The *Cs*caspase-1 transcripts were found in all tissues of the fifth instar larvae, and higher levels were found in the midgut, hindgut and Malpighian tubules. Examination of *Cs*caspase-1 expression in different developmental stages indicated low constitutive levels in the eggs and early larvae stages, and higher abundances were exhibited in the last larvae and pupae stages. The relative mRNA levels of *Cs*caspase-1 were induced by heat and cold temperatures. For example, the highest increase of *Cs*caspase-1 transcription was at −3 °C and 36 °C respectively. In a word, *Cs*caspase-1 plays a role of effector in the apoptosis of *C. suppressalis*. It also correlates with development, metamorphosis and thermotolerance of *C. suppreassalis*.

## 1. Introduction

Although apoptosis was first described in the publication over forty years ago [1], it still draws great attention as a result of its role in development, host defense, and a response of cells to general stress [2–5]. Apoptosis is executed through the activity of the caspases that are aspartyl-specific proteases [6,7]. The caspase family is divided into two groups: the initiators and the executors of apoptosis, each including three domains: an amino terminal domain, a large subunit and a small subunit [8]. In insects, caspases also play an important role in apoptosis [9]. The *Drosophila* genome contains seven caspase genes that function in both immune signaling pathways and development. In *Aedes aegypti*, *Aedes* Dronc, a kind of caspase gene, also possesses similar roles [10,11]. Five caspase family members were found in *Bombyx mori* genome [12]. 66 caspase sequences representing 27 lepidopteran insect species can be classified into at least five groups by phylogenetic analyses [13]. However, completed studies on the genome, structure and functions of caspases in insect were found infrequently, especially the relationship with thermotolerance [5,14–17].

The thermotolerant ability of insect can be divided into basal and inducible components [18]. When exposed to adverse temperatures, organisms may respond differently including the induction of caspases that causes the apoptosis. For example, low temperature led to apoptosis in some mammalian cells [19], and high temperature could significantly promote apoptosis process resulting in the increased mortality rate of *Plutella xylostella* [16]. The caspase is also related to the cold hardness in *Drosophila melanogaster* [5]. In order to understand the molecular mechanism of thermotolerance of insect, it’s necessary to study the relation between caspase and thermotolerance.

The striped stem borer, *Chilo suppressalis* (Walker) (Insecta: Lepidoptera: Pyralidae), is one of the most insect pests of rice in Asia, Northern Africa and Southern Europe, and has a complex life cycle. According to our studies in 2010, field-collected larvae in March could survive at −21 °C [20], and the field larvae in summer could tolerance 46 °C in Yangzhou area, Jiangsu province, China. In the present work, we set out to investigate and isolate caspase-1 in *C. suppressalis*. Hereafter, its structure model was predicted. At the same time, caspase-1 *m*RNA expression of different tissues (organs), developmental stages and temperatures was demonstrated.

## 2. Results and Discussion

### 2.1. Isolation, Cloning, Sequencing and Structure of Cscaspase-1

**Figure 1:**
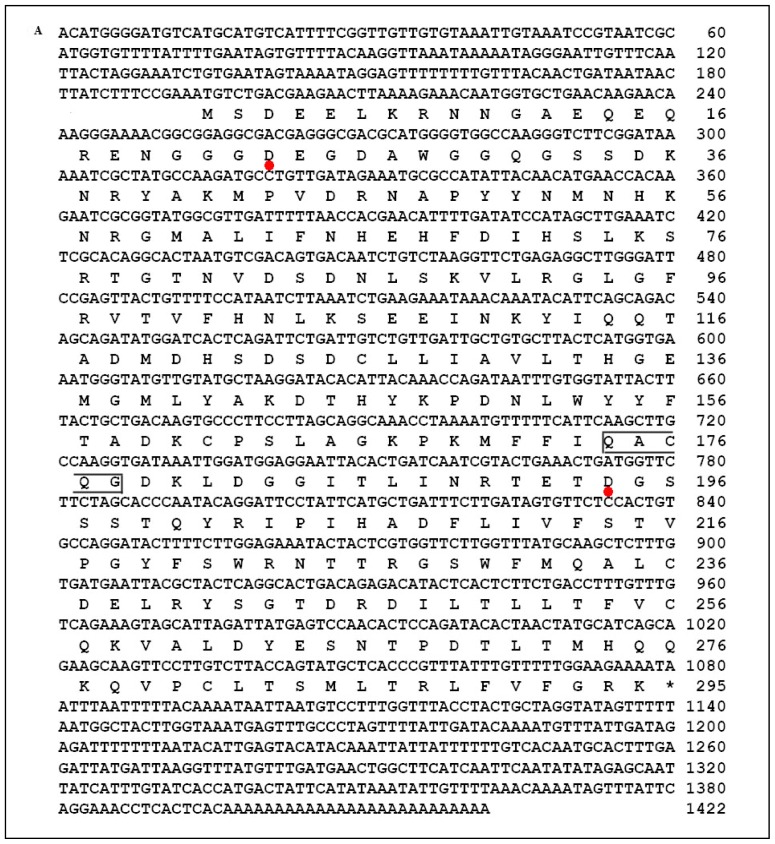

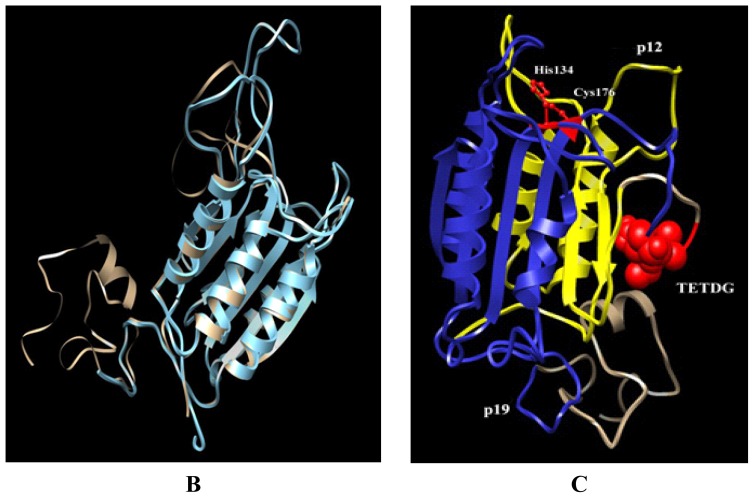
The sequence and structure analysis of the *Cs*caspase-1. (**A**) Nucleotide and deduced amino acid sequence of *Cs*caspase-1 (JQ864206). Two cleaved sites to generate the large and small subunit are also shown by red dots. The active site pentapeptide QACQG was boxed; (**B**) Homology modeling of the *Cs*caspase-1 (light yellow) with *Spodoptera frugiperda* caspase-1 (PDB ID: 2NN3) (cornflower blue) as template using Phyre2 software; (**C**) The large (p19) and small subunit (p12) regions respectively are shown in blue and red. TETDG motifs are shown as red spheres and two active sites (His^134^ and Cys^176^) are shown as red sticks.

Degenerate primers based on conserved regions from several insect caspase-1 were used to amplify a 359 bp partial fragment from *C. suppressalis* cDNA. The cloned fragment was sequenced and a BLAST analysis of its deduced amino acid fragment revealed apparent sequence homology with the caspase-1 (data not shown). The full-length 1422-bp *Cs*caspase-1, including the UTRs, was obtained through 5′ and 3′ RACE (GenBank accession no. JQ864206). It possesses the open reading frame (ORF) of 295 amino acids with a molecular weight of 33.56 kDa and an isoelectric point of 6.2. Two cleavage sites were found to be located between the prodomain and large subunit at Asp^23^, and between the large and small subunits at Asp^194^. Moreover, *Cs*caspase-1 contains QACQG pentapeptide active-site motif, which is also found in lepidopteran caspase-1 (Figure 1A) [5,14–17].

To investigate the potential structure-function relationship of *Cs*caspase-1, we generated its homology model with Phyre using *Spodoptera frugiperda* caspase-1 (PDB ID: 2NN3) as a template [14]. The structure of the *Cs*caspase-1 model was very similar to that of caspase-1 of *S. frugiperda* (confidence, 100% statistically and identity, 87% based on sequence alignment) (Figure 1B). The model shows that *Cs*caspase-1contains three predicted domains, a prodomain, a large subunit domain (p19), and a small subunit domain (p12). Two active sites were found to be located at His^134^ and Cys^176^ respectively, which is known to be involved in executing apoptosis (Figure 1A,C). Through cleavage sites, pro-*Cs*caspase-1 would be cleaved to the mature large subunit p19 and small subunit p12. Similar results were exhibited in *S. frugiperda* [14]. The *N*-terminal segment of the large subunit of *Cs*caspase-1 lies near the TETDG motifs, containing the activation cleavage site (Figure 1C). In a word, *Cs*caspase-1 plays a role of effector in the apoptosis of *C. suppressalis*.

### 2.2. Phylogenetic Analysis

**Figure 2:**
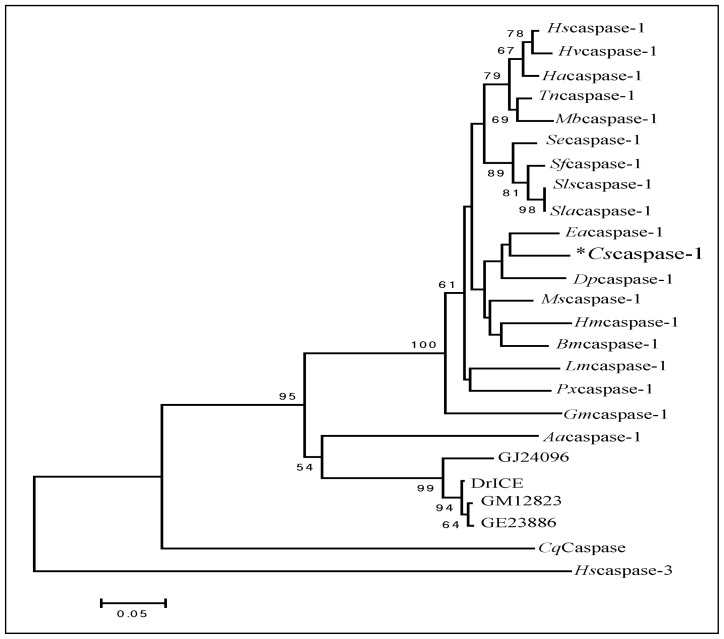
Neighbor-joining phylogenetic tree showing the *C. suppressalis* (*Cs*caspase-1, I2EC37) with respect to the other caspases. *Heliothis subflexa* (*Hs*caspase-1, G0XQE5), *Heliothis virescens* (*Hv*caspase-1, G0XQE6), *Helicoverpa armigera* (*Ha*caspase-1, A9QOJ3), *Trichoplusia ni* (*Tn*caspase-1, B6EEC1), *Mamestra brassicae* (*Mb*caspase-1, G0XQE8), *Spodoptera exigua* (*Se*caspase-1, G1ELA2), *Spodoptera frugiperda* (*Sf*caspase-1, P89116), *Spodoptera littoralis* (*Sls*caspase-1, Q8I955), *Spodoptera litura* (*Sla*caspase-1, H6V6K4 ), *Euphydryas aurinia* (*Ea*caspase-1, F6K5S3), *Danaus plexippus* (*Dp*caspase-1, G6DDV5), *Manduca sexta* (*Ms*caspase-1, F6K5R8), *Heliconius melpomene* (*Hm*caspase-1, C6YXH1), *Bombyx mori* (*Bm*caspase-1, H9JBQ1), *Lymantria monacha* (*Lm*caspase-1, G0XQE7), *Plutella xylostella* (*Px*caspase-1, D9IVD4), *Galleria mellonella* (*Gm*caspase-1, G0XQE4), *Aedes aegypti* (*Aa*caspase-1, Q16MZ1), GJ24096 (*Drosophila virilis*, B4JM0P7), DrICE (*Drosophila melanogaster*, O01382), GM12823 (*Drosophila sechellia*, B4HZF1), GE23886 (*Drosophila yakuba*, B4PPJ9), *Cq*caspase (*Culex quinquefasciatus*, B0WPG0), *Hs*caspase-3 (*Homo sapiens*, P42574). Numbers on the branches are the bootstrap values obtained from 1000 replicates (only bootstrap values >50 are shown).

We used CLUSTALX and MEGA 5.0 phylogenetic analysis to compare *Cs*caspase-1 with other caspases. Figure 2 illustrates the phylogenetic tree constructed by the neighbor joining method. The newly sequenced *C. suppressalis* is most closely related to *Ea*caspase-1, to which they are 85% identical at the amino acid level. The *Cs*caspase-1 shares 62% identity with DrICE (*Drosophila melanogaster*) and 38% identity with human caspase-3. The phylogenetic analysis of insect caspase-1 has revealed the divorce between Lepidoptera and Diptera. Moreover, in Lepidoptera, all months of Noctuidae fell into the well-supported cluster (Figure 2).

### 2.3. Genomic Analysis of Cscaspase-1

**Figure 3:**
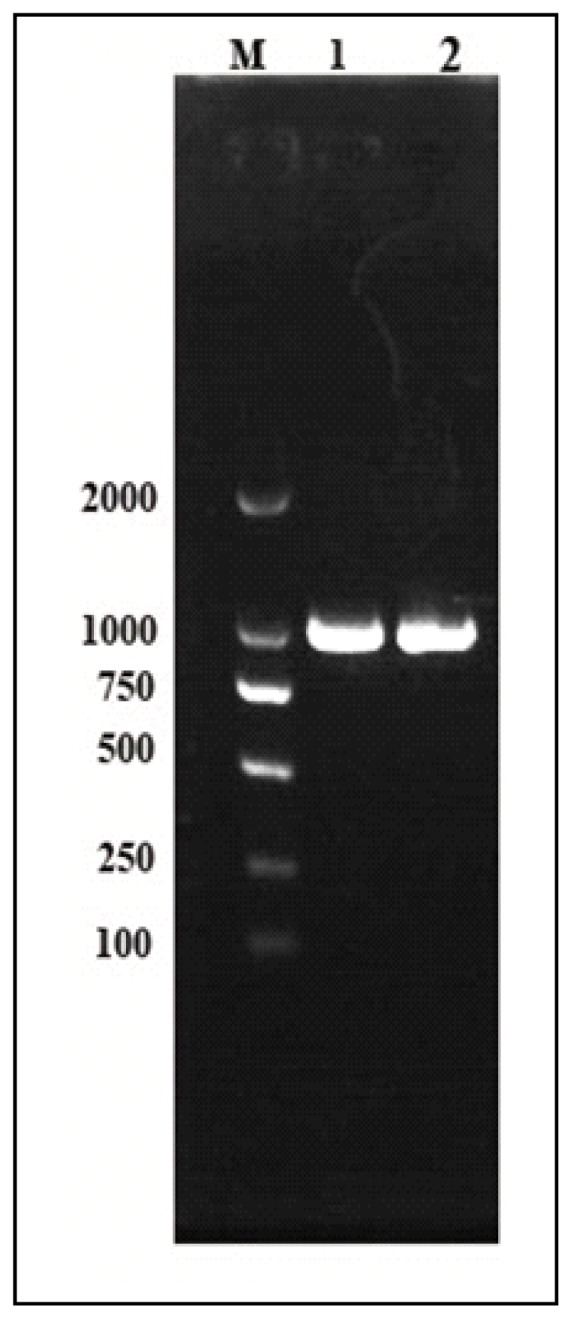
The PCR products of *Cs*caspase-1 DNA in the agarose gel electrophoresis (1.0%). Lane M contains 2 kb DNA molecular weight, lanes 1 and 2: the PCR product of *Cs*caspase-1 DNA, respectively.

A pair of *Cscaspase-1* specific primers was used to amplify a 985 bp DNA fragment from *C. suppressalis* (Figure 3). Hereafter the PCR products were sequenced. Comparative alignment of the genomic sequence with corresponding cDNA sequence showed that there was no intron in the genome of *Cscaspase-1*. The negative correlation between intron size and gene expression level was suggested in some studies, and the shorter or no introns genes had highly expressed level [21–23]. However, apoptosis is very important to lead to various biological processes, such as development [24], tissue homeostasis, DNA damage response [25] and stress response [5]. So, the *Cscaspase-1* didn’t have any intron, in order to need to highly express in various biological processes, on the basis of the least costs of transcription.

### 2.4. Tissues (organs) Distribution of Cscaspase-1mRNA Expression in *C. suppressalis* Larvae

**Figure 4:**
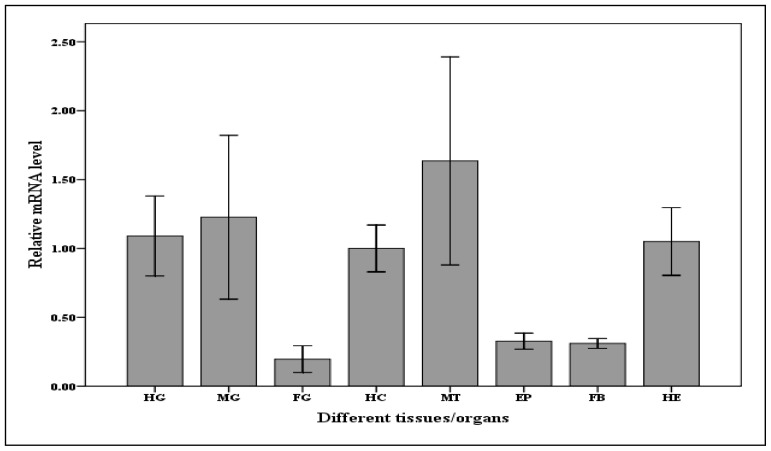
Relative mRNA expression levels of the *Cs*caspase-1 gene in different tissues (organs) of the *C. suppressalis* larvae. Abbreviation: HG, hindgut, MG, midgut, FG, foregut, HC, haemocytes, MT, Malpighian tubules, EP, epidermis, FB, fat body, HE, Head. Values are denoted as mean ± SE. The quantity of each *Cs*caspase-1 mRNA is normalized to the abundance of 18SrRNA.

The *Cs*caspase-1 transcripts were found in all tissues of the fifth instar larvae screened in this study, and the higher levels were found in the midgut, hindgut and Malpighian tubules. Interestingly a high expression level was also detected in hemocytes (Figure 4). Similar results were found in *Galleria mellonella* and *Helicoverpa armigera* [15,17]. Hindgut and Malpighian tubules reabsorb water, salts, and other substances before excretion in insect. However, some toxic substances, such as uric acid, could induce the apoptosis of cell [26]. The larval midgut epithelium of Lepidoptera degenerates during metamorphosis [27–30]. Since apoptosis plays a central role in tissue remodeling, we would expect to detect higher basal levels of caspases in tissues with rapid renewal rates. The haemolymph in which all the internal organs are bathed might migrate the *Cs*caspase-1 to any organ had to execute the apoptosis.

### 2.5. Expression of Cscaspase-1 in the Developmental Stages of the *C. suppressalis*

**Figure 5:**
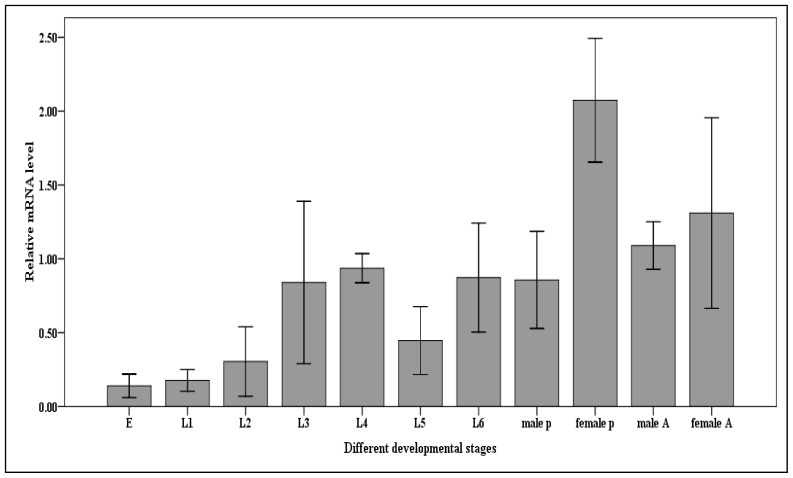
Relative mRNA expression levels of the *Cs*caspase-1 gene in different developmental stages of the *C. suppressalis*. Abbreviation: E, eggs, L1, the first instar larvae, L2, the second instar larvae, L3, the third instar larvae, L4, the fourth instar larvae, L5, the fifth instar larvae, L6, the sixth instar larvae, P, pupae, A, Adults. Values are denoted as mean ± SE. The quantity of *Cs*caspase-1 mRNA is normalized to the abundance of 18SrRNA.

In order to determine *Cs*caspase-1 exhibit developmental expression patterns, we analyzed basal expression in the eggs, first, second, third, fourth, fifth, sixth instar larvae, pupae and adults of *C. suppressalis*. The results demonstrated unexpectedly the lowest expression level of *Cs*caspase-1 was observed in the eggs. Then the level was increased by the development of *C. suppressalis*. The most abundance was detected in female pupae. The results also profiled the *Cs*caspase-1 mRNA had a higher level in female pupae than those of male (Figure 5). It is obvious when looking at a mature organism that it is the product of a series of cellular production and degradation events. Especially, the development of holometabolous insects is characterized by a complete metamorphosis. Examination of *Cs*caspase-1 expression in different developmental stages indicated low constitutive levels in the eggs and early larvae stage. In insects, the steroid hormone ecdysone regulates the genes of caspases [11,15,31,32]. The growth and metamorphosis of insects from embryo to adult, including each larval molting, are triggered by pulses of ecdysone. The similar results showed two ecdysone pulses happened respectively at the end of third larval instar and early pupal stage in *Drosophila* [33–35]. Totally, we concluded that the *Cs*caspase-1 could contribute to the development of *C. suppressalis*.

### 2.6. Expression of Cscaspase-1 mRNA under Various Temperatures

**Figure 6:**
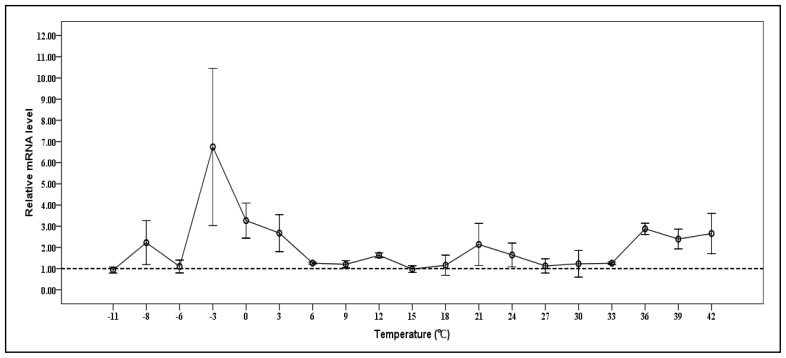
Relative expression levels of the *Cs*caspase-1 in the rice stem borer under temperature stress. The larvae was exposed for −11 °C, −8 °C, −6 °C, −3 °C, 0 °C, 3 °C, 6 °C, 9 °C, 12 °C, 15 °C, 18 °C, 21 °C, 24 °C, 27 °C, 30 °C, 33 °C, 36 °C, 39 °C and 42 °C for 2 h. Values are denoted as mean ± SE. The quantity of each *Cs*caspase-1 mRNA is normalized to the abundance of 18SrRNA.

In present work, the relative mRNA levels of *Cs*caspase-1 of *C. suppressalis* were tested at temperature gradients from −11 °C to 42 °C. The level was increased in larvae treated at some temperatures, and the highest level that were reached to 6.7 fold as compared to that of control temperature was observed in larvae exposed at −3 °C, 2 h. During heat stress, the *Cs*caspase-1 level reached maximum level at 36 °C, which increased 2.9 fold of control. There were different expression trends between high and low temperature in this study. For example, the mRNA level of *Cs*caspase-1 under low temperature fluctuated strongly comparing to the heat (Figure 6). Our studies showed that the *Cs*caspase-1 transcript can be upregulated under heat and cold stress. The two highest increases were at −3 °C and 36 °C. In *C. suppressalis*, *Cs*caspase-1 could be coordinated to encounter with the following temperature stress. When the specimens are exposed to extreme temperature, some cells can produce the apoptosis in order to decrease the cost of survival.

## 3. Experimental Section

### 3.1. Insects

The population of *C. suppressalis* was collected from the suburb of Yangzhou (32.39°N, 119.42°E). The rice striped borers were reared in an environmental chamber at 28 ± 1 °C, 16:8 (L:D) and RH = 70% ± 5% [36].

### 3.2. Cloning and RACEs

Total RNA was extracted by the SV Total RNA isolation system (Promega Z3100) combined with DNase digestion to eliminate DNA contamination. Total cDNA was synthesized by oligo(dT)18 primer (TaKaRa). The full-length cDNA of the caspase-1 gene was determined using 5′- and 3′-RACE (SMART RACE, Clontech). The primers used are shown in Table 1. To ensure that the 5′ and 3′ fragments were from the same gene, specific primer were designed, then used to PCR amplify the sequences.

### 3.3. Characterization of the Cscaspase-1 Genome

The genomic DNA of *C. suppressalis* was extracted by Axyprep^TM^ multisource Genomic DNA Kit (Axygen, USA). A pair of specific primers (GSP-1 and GSP-2) flanking the ORF was designed to amplified *Cs*caspase-1 genomic fragment (Table 1). A touch-down PCR was used, and the parameters are as follows: 94 °C for 5 min, 15 cycles at 94 °C for 30 s, 65–50 °C (decreasing by −1 °C/cycle) for 30 s, and 72 °C for 1 min 30 s, followed by 30 cycles of 94 °C for 30 s, 50 °C for 30 s, and 1 min 30 s, and a final extension at 72 °C for 10 min. Hereafter the PCR amplified fragment was sequenced.

### 3.4. Sample Preparation

The rice striped borers were reared successively to third generation in the seedlings. Then, the egg masses, the first, second, fourth, fifth, sixth instar larvae, pupae (male and female), and one-day adults (male and female), were randomly selected for the experiment. Each experiment was repeated three times. The fifth instar larvae were anesthetized on ice for 15 min before dissection. Head (HE), epidermis (EP), fat body (FB), foregut (FG), midgut (MG), hindgut (HG), Malpighian tubules (MT), and haemocytes (HC) were collected and rinsed with 0.9% sodium chloride solution. The samples were frozen immediately in liquid nitrogen and stored at −80 °C until the experiment.

### 3.5. Temperature Stress

Larvae used in experiments were all 5th instars of similar body size and were assigned randomly to each experimental group. Each experimental group contained 10 larvae; each larva was confined individually in glass tubes and exposed to a given temperature (including −11 °C, −8 °C, −6 °C, −3 °C, 0 °C, 3 °C, 6 °C, 9 °C, 12 °C, 15 °C, 18 °C, 21 °C, 24 °C, 27 °C, 30 °C, 33 °C, 36 °C, 39 °C, and 42 °C) for 2 h in a constant temperature subzero incubator (DC-3010, Jiangnan equipment). The larvae were recovered for 2 h. Survived larvae (Table S1) were frozen in liquid nitrogen and then stored at −70 °C. Each treatment included more than three individual larvae.

### 3.6. Quantitative Real-time PCR (qPCR) Analysis

Total RNA was extracted by the SV Total RNA isolation system (Promega Z3100), followed by DNase treatment to eliminate DNA contamination. The reaction volume was 20 μL. Each reaction contained 10 μL of 2× SYBR^®^Premix EXTaqTM (TaKaRa, Dalian, China) master mix, 0.8 μL of each of gene specific primers (caspase-1-F and caspase-1-R) (Table 1), 0.4 μL of Rox reference Dye, and 2 μL of cDNA templates. The annealing temperature was 62.9 °C. Reactions were carried out on a CFX-96 real-time PCR system (Bio-Rad). The efficiencies of the target and reference genes are similar. The quantity of *Cs*caspase-1 mRNA was calculated using the 2^−ΔΔCt^ method [37], and normalized to the abundance of the 18SrRNA gene. Following qPCR, the homogeneity of the PCR products was confirmed by melting curve analysis.

### 3.7. Bioinformatic Analysis

The open reading frames (ORFs) were identified with the aid of the ORF Finder software [38]. The deduced amino acid sequences were aligned using ClustalX software. Sequence analysis tools of the ExPASy Molecular Biology Server of Swiss Institute of Bioinformatics, including Translate, Compute pI/MW and Blast were used to analyze the deduced caspase-1 protein sequence. Amino acid sequences were used to estimate phylogeny with the neighbor-joining, minimum evolution, maximum likelihood and maximum parsimony methods. Phylogenetic trees were constructed with 1000 bootstrap replicates using MEGA version 5.0 [39].

### 3.8. Computational Molecular Modeling

Homology models were generated using Protein Homology/analogy recognition engine V 2.0 [40]. Briefly, the caspase-1 sequence was aligned by the Phyre2, and the best model of *Spodoptera frugiperda* X-ray D* structure (PDB ID: 2NN3) was used for modeling analyses. The Chimera Tool was used to visualize the three-dimensional coordinates for the atoms of the model [41].

## 4. Conclusions

In this study, we successfully isolated and characterized *Cs*caspase-1 of *C. suppressalis. Cs*caspase-1 provides us with the opportunity for a study on the role of caspase family in development, metamorphosis and the role of apoptosis in regulating thermotolerance of insect. However, regulations of caspases in the apoptosis were a highly complex and dynamic process. Therefore, we are undertaking characterizations and further analysis of other caspases in *C. suppressalis*, including initiator and effector. Caspase cleavage is required for activation, while induction of mRNA does not completely indicate activation. Thus, we plan further study on the cleavage activation of *Cs*caspase-1. We expect that investigations of caspases will play key roles in the integrated management of *C. suppressali* in the future.

## Supplementary Information



## Figures and Tables

**Table 1:** Primers used in the cDNA cloning, DNA analysis and real-time quantitative PCR.

Primers	Sequences (5′–3′)
DP-F	AYCAYGARCATTTYGAHATTCACA
DP-R	DCCACCATCCRATTTATCACCTTG
caspase-1 5′	CCTGTGCGAGATTTCAAGCTGTGA
caspase-1 3′	ACTTTACTGCTGACAAGTGCCCTTCC
caspase-1-F	TGCATGTCATTTTCGGTTGTTG
caspase-1-R	CTCCGCCGTTTTCCCTTTG
GSP-1	CGAAATGTCTGACGAAG
GSP-2	AAACTAGGGCAAACTCA
18SrRNA-F	CACGGGAAATCTCACCAGG
18SrRNA-R	CAGACAAATCGCTCCACCAACTA
UPM(Long)	CTAATACGACTCACTATAGGGCAAGCAGTGGTATCAACGCAGAGT
UPM(Short)	CTAATACGACTCACTATAGGGC

Note: DP, degenerate primers and UPM, universal primer mix.
