# Supplementary material for: Molecular Cloning and Characterization of the First Caspase in the Striped Stem Borer Chilo suppressalis
Source: Int J Mol Sci. 2013 May 15;14(5):10229–41. doi: 10.3390/ijms140510229 (PMC3676837; doi:10.3390/ijms140510229)
Supplement: Supplementary file 1 [file ijms-14-10229-s001.pdf]

Supplementary Information

Table S1. The survival rate of *C. suppressalis* under different temperatures.

Temperature treat (°C)	Total number	Survival number	Survival rate (%)
−11	9	5	55.56%
−8	10	9	90.00%
−6	9	9	100.00%
−3	10	10	100.00%
0	10	9	90.00%
3	10	8	80.00%
6	10	10	100.00%
9	10	10	100.00%
12	9	9	100.00%
15	10	10	100.00%
18	10	10	100.00%
21	10	10	100.00%
24	9	9	100.00%
27	10	10	100.00%
30	10	10	100.00%
33	10	10	100.00%
36	10	10	100.00%
39	10	10	100.00%
42	10	10	100.00%
45	10	0	0.00%

© 2013 by the authors; licensee MDPI, Basel, Switzerland. This article is an open access article distributed under the terms and conditions of the Creative Commons Attribution license (http://creativecommons.org/licenses/by/3.0/).
